# Study Protocol: Connecting older adults through multi-level programmes for alleviating loneliness in Hong Kong older adults (COMPASS-HOA)

**DOI:** 10.3389/fpubh.2026.1741422

**Published:** 2026-02-23

**Authors:** Vivien Foong Yee Tang, Da Jiang, Jojo Yan Yan Kwok, Henry C. Y. Ho, Izzy Yi Jian, Namkee G. Choi, Tegan Cruwys, Joyce Hoi Sze You, Lisa M. Warner, Noëmi Seewer, Xue Bai, Anton Käll, Gerhard Andersson, Tobias Krieger, Brynmor Lloyd-Evans, Kee Lee Chou

**Affiliations:** 1 Department of Social Sciences and Policy Studies, The Education University of Hong Kong, Hong Kong, Hong Kong SAR, China; 2 Department of Special Education and Counselling, The Education University of Hong Kong, Hong Kong, Hong Kong SAR, China; 3 School of Nursing, The University of Hong Kong, Hong Kong, Hong Kong SAR, China; 4 Centre on Behavioral Health, Faculty of Social Sciences, The University of Hong Kong, Hong Kong, Hong Kong SAR, China; 5 Department of Psychology, The Education University of Hong Kong, Hong Kong, Hong Kong SAR, China; 6 School of Social Work, University of Texas at Austin, Austin, TX, United States; 7 School of Medicine and Psychology, Australian National University, Canberra, ACT, Australia; 8 School of Pharmacy, The Chinese University of Hong Kong, Hong Kong, Hong Kong SAR, China; 9 Department of Psychology, MSB Medical School Berlin, Berlin, Germany; 10 Department of Clinical Psychology and Psychotherapy, University of Bern, Bern, Switzerland; 11 Department of Applied Social Sciences, The Hong Kong Polytechnic University, Hong Kong, Hong Kong SAR, China; 12 Department of Behavioral Sciences and Learning, Linköping University, Linköping, Sweden; 13 Department of Biomedical and Clinical Sciences, Linköping University, Linköping, Sweden; 14 Division of Psychiatry, University College London, London, United Kingdom

**Keywords:** cluster randomized controlled trial, cognitive behavioral treatment, Groups 4 Health, loneliness, mindfulness, multi-level intervention, neighbours every day

## Abstract

**Background:**

Loneliness is a widespread global issue among older adults that negatively impacts physical and mental health, cognitive functioning, quality of life, and longevity, while also burdening society with healthcare costs. Resulting from factors at individual, interpersonal, and community levels. Multi-level interventions targeting these levels show promise in addressing this complex issue.

**Objective:**

This trial aims to develop, implement, and evaluate a multi-level intervention comprising individual-, interpersonal-, and community-level components to reduce loneliness and enhance well-being among older adults in Hong Kong.

**Methods:**

This pragmatic, assessor-blinded, 4-arm cluster randomized controlled trial will evaluate a multi-level intervention among 1,344 community-dwelling lonely older adults living in poverty. Participants will be randomized equally to one of the four 6-month intervention groups delivered by trained volunteers: individual-level only, individual- + interpersonal-level, and individual- + interpersonal- + community-level interventions against an education-only control group. Data will be collected at six time points: baseline; 2, 4, and 6 months post baseline; and 12 and 24 months after intervention completion. Primary outcome is assessed by the Revised UCLA Loneliness Scale. Secondary outcomes include De Jong Gierveld Loneliness Scale, symptoms of anxiety and depression, perceived stress, life satisfaction, psychological well-being and sleep quality. Potential mediators will be assessed by mindfulness, self-compassion, emotion regulation, perceived social support, social network characteristics, and neighbourhood collective efficacy and identification. Analyses will follow the intention-to-treat principle, employing linear mixed-effects models, regression, mediation, and cost-effectiveness. Qualitative data collected via focus group discussions will be analysed thematically to gain deeper insight into participants’ experiences and perceptions of the different interventions.

**Results:**

This study will last for 4 years, starting from June 2025 to May 2029. Recruitment and enrolment will begin in March 2026. Data collection will commence in April 2026, followed by the start of intervention delivery.

**Conclusion:**

To our knowledge, this is the first study globally to implement a multi-level intervention targeting older adults experiencing loneliness and poverty. If proven effective, the multi-level intervention will establish a foundation for community-wide implementation, providing a timely, affordable, and scalable approach that leverages community social capital.

**Clinical trial registration:**

ClinicalTrials.gov, identifier NCT07123064.

## Introduction

Loneliness, defined as the subjective perception of a gap between desired and actual social connections (1), has emerged as a critical public health challenge (2). In Hong Kong, approximately 46% of older adults report experiencing loneliness (3), a prevalence shaped by age-related health decline, reduced mobility, and economic vulnerabilities (4, 5). Loneliness is associated with adverse physical and mental health outcomes and increased healthcare utilization, underscoring its importance as both social and health concern (6–12).

This issue is intensified among older adults living in poverty. Nearly 45% of Hong Kong’s aging population lives in poverty, largely due to demographic changes and inadequate retirement protections (13). Economic vulnerability not only threatens older adults’ financial security but also heightens their risk of loneliness by limiting access to social participation and community resources (3). Although loneliness affects individual across all socioeconomic backgrounds, interventions that specifically target economically disadvantaged older adults are essential for addressing health inequalities and addressing the compounded social and health challenges they face (14, 15). Therefore, it is imperative to develop effective and scalable interventions for this population.

Despite numerous efforts to mitigate loneliness among older adults, the most effective intervention remains undetermined (16–19). Loneliness arises from a complex interplay of factors spanning from individual, interpersonal, and community factors (20, 21). Increasingly, systematic reviews and meta-analyses suggest that interventions addressing multiple levels simultaneously may be more effective than single-level approaches, as they leverage on reinforcing mechanisms across social contexts (17–19, 22, 23). However, multi-level interventions are often complex, resource-intensive, and robust trials evaluating their incremental benefits and cost-effectiveness remain scarce. To our knowledge, no randomized trial has systematically integrated the individual-, interpersonal-, and community-level components to address loneliness among older adults.

Against this backdrop, the present study aims to address this gap by evaluating the incremental benefits of a multi-level interventions. Specifically, we examine whether augmenting an individual-level intervention with interpersonal and community components leads to greater and more sustained reduction in loneliness.

At the individual level, psychological interventions have shown promising results in reducing loneliness (24, 25). Mindfulness-based approaches foster present-moment awareness and emotional regulation (26, 27), while behavioral activation promotes engagement in meaningful and rewarding activities; both have shown effectiveness in alleviating loneliness (25, 28). In Hong Kong, these approaches were successfully adapted for telephone delivery by trained volunteers during the COVID-19 pandemic (29, 30, 96), although their effectiveness beyond pandemic remains uncertain. Meta-analyses further indicate that interventions combining cognitive behavioral therapy (CBT) with strategies to enhance social connection are among the most promising strategies for reducing loneliness (17, 19). CBT enables older adults to modify maladaptive thoughts and behaviors related to themselves and social situations (31). Supporting this, an RCT demonstrated that an eight-week internet-based CBT (ICBT) intervention incorporating functional behavioral model, cognitive restructuring, behavioral activation to increase social participation, and psychoeducation effectively reduced loneliness in adults (32). Drawing on this evidence, we will enhance our individual-level intervention by integrating a cognitive component adapted from the ICBT developed by Käll et al. (32), with a locally validated mindfulness-based intervention (29, 30, 96), delivered by trained volunteers via the telephone, a modality selected for its accessibility, low cost, and feasibility among older adults facing mobility, digital, or socioeconomic barriers.

At the interpersonal level, interventions that strengthen social networks and enhance relationship quality play a central role in mitigating loneliness, particular among socially isolated individuals (33). These approaches focus on identifying meaningful social ties, rebuilding existing relationships, and fostering new connections through structured engagement (34, 35). Interventions like Groups 4 Health (G4H) (36) and Community Navigator (37), which emphasize social group belonging, have demonstrated efficacy in reducing loneliness among university students and adults with anxiety or depression. G4H was selected as the foundation for this level because it is explicitly designed to enhance social group identification and belonging—a key protective mechanism against loneliness. Importantly, G4H has also been adapted and piloted in older adults, yielding promising improvements in social cohesion, social group connections, depressive symptoms, and overall well-being (38). Although social isolation and loneliness are distinct constructs, social isolation remains a significant risk factor for loneliness. Accordingly, we will implement an interpersonal-level intervention, by adapting and modifying the Groups 4 Health (39) programme to the local context, delivered via telephone, and facilitated by trained volunteers.

At the community level, social infrastructure and neighbourhood cohesion play a central role in mitigating loneliness. Access to local social resources and supportive neighbourhood environments protects individuals at risk of loneliness (40, 41). Evidence suggests that interactions with neighbours may confer greater benefits for mental, cognitive, and physical health—lower mortality risk—than family engagement alone (23). In Hong Kong, neighbourhood collective efficacy has been identified as a protective factor that buffers the adverse effects of poverty on loneliness (3). Community-based, event-driven community interventions have also demonstrated effective in improving older adults’ mental well-being by strengthening neighbourhood connections and fostering social cohesive environment (42). Since its launch in 2003, the Neighbours Every Day (NED) programme in Australia has successfully promoted neighborly engagement through locally organized events, leading to sustained improvements in neighbourhood affinity, social cohesion, and reductions in loneliness (43). These benefits persisted for at least 6 months, with participants reporting stronger neighbourhood ties and larger social networks than non-participants (44, 45). Thus, our community-level intervention will implement in-person, community-led events modeled after the Neighbours Every Day programme.

**Figure 1:**
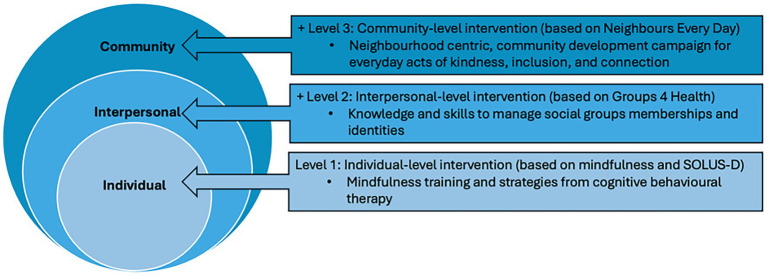
Illustration of multi-level interventions.

Notably, there is a lack of longitudinal data on the sustained effects of loneliness interventions on health, cognition, and healthcare utilization (46). To address this gap, the present study will conduct follow-up assessments over 2 years to compare the effectiveness of the individual only, individual + interpersonal, and individual + interpersonal + community interventions against an education-only control group (Figure 1). In addition, we will examine the mechanisms through which these interventions exert their effects and assess their cost-effectiveness to inform resource allocation. This study will clarify not only the comparative effectiveness of each intervention level but also the underlying mechanisms and cost-effectiveness, providing essential evidence for the scalable implementation of community-based programmes.

### Objectives

This primary objective of this study is to develop, implement, and evaluate a multi-level intervention comprising individual-, interpersonal-, and community-level components to reduce loneliness and enhance well-being among older adults in Hong Kong. The specific goals are to:

1. Evaluate the short-term effectiveness of the individual-level intervention, compared with an education-only control group in reducing loneliness.
2. Assess the incremental benefit of adding an interpersonal-level intervention to the individual-level intervention in further reducing loneliness, compared with the individual-level intervention alone.
3. Assess the incremental benefit of adding a community-level intervention to the combined individual- and interpersonal-level intervention in reducing loneliness.
4. Examine potential mediators through which individual-, interpersonal-, and community-level interventions influence loneliness outcomes.
5. Examine the long-term effects of the individual-level only, individual- + interpersonal-level; and individual- + interpersonal- + community-level interventions, compared with an education-only control group, on loneliness, physical and mental health, cognitive functioning, and healthcare utilization.
6. Evaluate the cost-effectiveness of individual-level only, individual- + interpersonal-level; and individual- + interpersonal- + community-level interventions compared with education-only control group.

## Methods

### Study design

**Figure 2:**
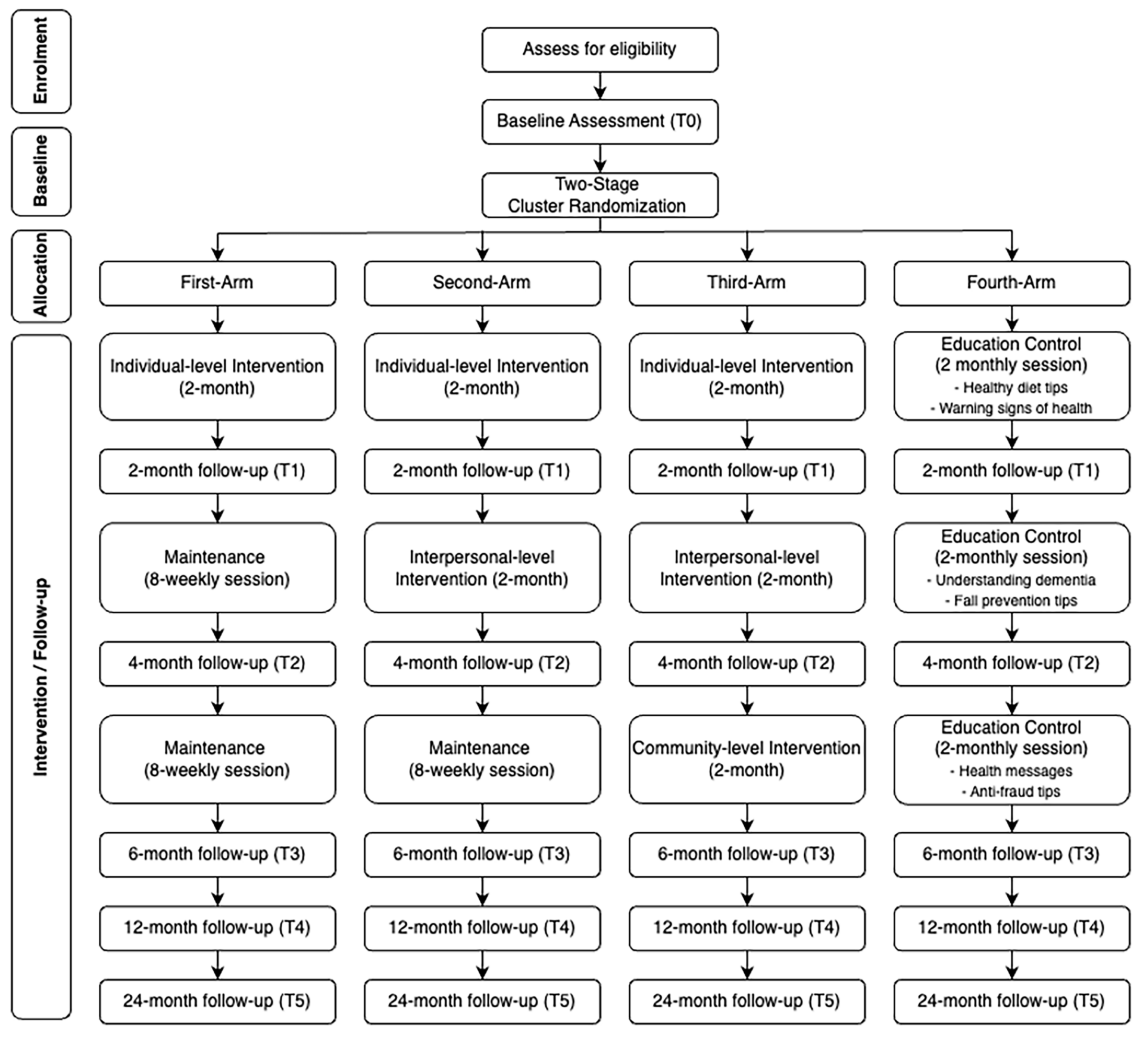
CONSORT flow diagram.

This study will employ a single-blinded, four-arm cluster randomized controlled trial to evaluate the effectiveness of reducing loneliness of an (i) individual-level only, (ii) individual- + interpersonal-level, (iii) individual- + interpersonal- + community-level interventions, compared to (iv) an education-only control group. This study will span across 4 years, commencing June 2025 to May 2029, to allow sufficient time for recruitment, intervention delivery, and both short- and long-term follow-up assessments. Figure 2 shows the CONSORT flow diagram. All study procedures will strictly adhere to the SPIRIT guidelines (Standard Protocol Items: Recommendations for Interventional Trials) as detailed in Table 1 (47).

**Table 1:** Schedule of enrolment, interventions, and assessments for SPIRIT 2025.

	Study period						
	Enrolment		Post-randomization				Close-out
Timepoint	−*t*_1_	*t*_0_	*t*_1_	*t*_2_	*t*_3_	*t*_4_	*t*_5_
Enrolment							
Eligibility screen	×						
Informed consent	×						
Randomization		×					
Interventions							
First-arm (individual-level only)			×				
Second-arm (individual- + interpersonal-level)			×	×			
Third-arm (individual- + interpersonal- + community-level)			×	×	×		
Fourth-arm (education-only control group)			×	×	×		
Maintenance				×	×		
Assessments							
Primary outcomes							
Revised UCLA Loneliness Scale (59, 60)		×	×	×	×	×	×
Secondary outcomes							
De Jong Gierveld 6-item Loneliness Scale (61, 62)		×	×	×	×	×	×
Pittsburgh Sleep Quality Index (63, 64)		×	×	×	×	×	×
Satisfaction with Life Scale (65, 66)		×	×	×	×	×	×
Activities of daily living (73)		×				×	×
Instructional activities of daily living (74, 75)		×				×	×
Physical Symptoms Inventory (76, 77)		×	×	×	×	×	×
Charlson Comorbidity Index (78)		×				×	×
6-Minute Walking Test (79, 80)		×				×	×
Perceived Stress Scale (67, 68)		×	×	×	×	×	×
Hospital Anxiety and Depression Scale (69, 70)		×	×	×	×	×	×
Hong Kong Montreal Cognitive Assessment (71, 72)		×				×	×
Mediators							
Mindfulness Attention Awareness Scale (81)		×	×	×	×	×	×
Self-Compassion Scale-Short Form (82, 83)		×	×	×	×	×	×
Emotion Regulation Scale-Cognitive Reappraisal subscale (84, 85)		×	×	×	×	×	×
Multidimensional Scale of Perceived Social Support (86, 87)		×	×	×	×	×	×
Lubben Social Network Scale (88, 89)		×	×	×	×	×	×
Neighbourhood Collective Efficacy Scale (90, 91)		×	×	×	×	×	×
Cost-effectiveness							
Quality of Life Measures (92, 93)		×	×	×	×	×	×
Use of healthcare services		×	×	×	×	×	×
Demographics							
Age, gender, number of children, education level		×					
Marital status, living with family members		×				×	×
Economic activity status, house		×	×	×	×	×	×
Usage of senior citizen center or service		×	×	×	×	×	×
Usage of community/Home care services		×	×	×	×	×	×
Household income in the past month		×	×	×	×	×	×

### Public housing in Hong Kong

Public housing system in Hong Kong is a cornerstone of the city’s housing policy, managed by the Hong Kong Housing Authority and the Hong Kong Housing Society, and primarily serve low-income households. There are approximately 260 public housing estates across 18 districts, providing a suitable setting for identifying and recruiting economically disadvantaged older adults (48). Eligibility for public rental housing is determined by means testing and residency requirement.

### Sampling and two-stage cluster randomization

Our study will employ a cluster randomized controlled trial (cRCT) design, with public housing estates as the units of randomization. A cRCT was selected because the community-level intervention is delivered at the cluster-level and is likely to influence social interactions among residents within the same estate, making individual randomization impractical and increasing the risk of contamination. All individuals within each cluster will receive the same intervention, outcomes will be measured at the individual level, and analyses will account for clustering effects (49).

**Figure 3:**
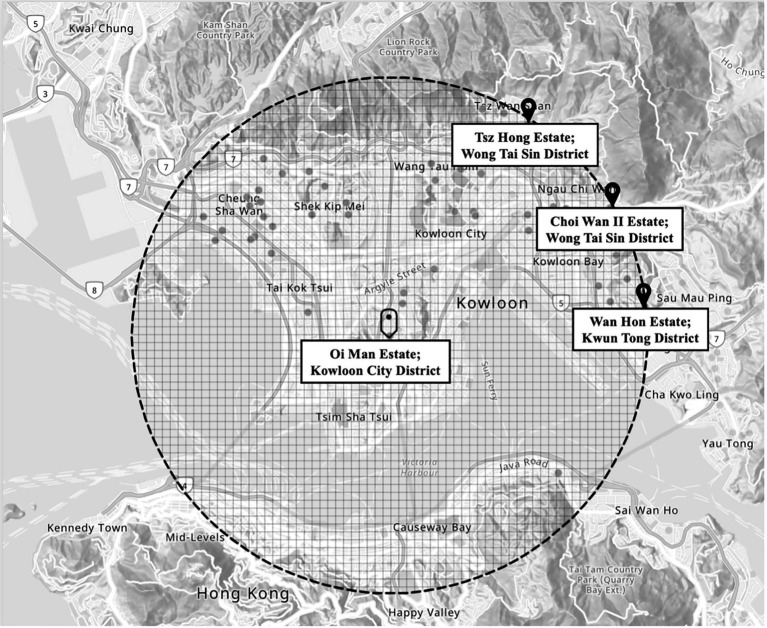
Map from GeoInfo Map (https://www.map.gov.hk/gm/), Lands Department, The Government of the Hong Kong Special Administrative Region.

A two-stage cluster-random sampling approach will be used. In the first stage, 32 clusters will be randomly selected from the 260 public rental housing estates across Hong Kong and assigned to one of the four intervention arms by an independent researcher using *Random.org* . The number of clusters may be adjusted if recruitment feasibility or cluster sizes vary. To minimize contamination, public rental housing estates assigned to different study arms will be geographically separated by at least 5 km, with a reasonable walking time of approximately 30 min or more. Figure 3 illustrates several estates located at least 5 km away from Oi Man estate, situated in the Kowloon City District. These estates include Tsz Hong Estate and Choi Wan II Estate in Wong Tai Sin District, as well as Wan Hon Estate in Kwun Tong District, with a walking time of at least 85 min. This spatial separation strategy is designed to reduce interaction between participants from different intervention arms, thereby maintaining the integrity of our intervention effects. In the second-stage, public rental housing units within each selected estate will be randomly sampled, and eligible residents will be invited to participate. This two-stage approach ensures an unbiased representation of older adults within each estates, while accounting for variation in cluster size.

### Sample size estimation

Based on a previous cluster randomized controlled trial designed to detect a difference between control and intervention groups for a continuous outcome (i.e., UCLA Loneliness Scale), an estimated variance was calculated at a two-tailed 5% level of significance, power of 95%, targeting a 0.087 unit difference on the UCLA Loneliness Scale while maintaining a relative precision of ±3.0% (49–52). Taking into account a 15% attrition rate across 24 months (53), unequal cluster sizes, intervention non-adherence, and potential ICC variability of *ρ* = 0.028, an effective sample size of 1,344 participants will be required, with 336 participants in each arm.

### Participants

The inclusion criteria are (i) Chinese older adults aged 65 years and older residing in a public housing estate, (ii) ability to complete the intervention in Cantonese, (iii) living in poverty, defined by self-reported material deprivation, operationalized as the inability to afford at least 5 items on the material deprivation index (54), and (iv) experiencing loneliness, defined as a score of ≥6 on the 3-item UCLA Loneliness scale (55). The exclusion criteria include (i) diagnosed with cognitive impairment, psychiatric disorder, learning disability, or suicidal ideation, and (ii) actively engaged in psychotherapy or psychosocial intervention in the past year.

### Allocation concealment and blinding

Randomization will be conducted by an independent researcher, and allocation will remain concealed from other researchers and participants until assignment. Outcome assessors will be blinded to participants’ intervention allocation. Participants will be partially unblinded, as they are aware of the intervention they receive, but they will be blinded to study hypotheses. Interventionists will be unblinded due to the nature of the interventions. To further reduce contamination, participants will be asked not to discuss the intervention with residents from other estates during the study period.

### Intervention

#### Structure

The intervention period will last for 6 months, with each intervention component delivered over a standardized two-month period (see Table 2). Participants will be allocated to one of the four study arms:

- First-arm (individual-level only):

**Table 2:** Intervention design.

Month	1				2				3				4				5				6			
Week	1	2	3	4	5	6	7	8	9	10	11	12	13	14	15	16	17	18	19	20	21	22	23	24
First-arm	**Individual-level only**																							
	Individual-level (Duration: once a week, 60 min per session, via telephone)								Individual-level maintenance (Duration: once a week, 15 min per session, via telephone)															
Second-arm	**Individual- + interpersonal-level**																							
	Individual-level (Duration: once a week, 60 min per session, via telephone)								Interpersonal-level (Duration: once a week, 60 min per session, via telephone)								Individual- + interpersonal-level maintenance (Duration: once a week, 15 min per session, via telephone)							
Third-arm	**Individual- + interpersonal- + community-level**																							
	Individual-level (Duration: once a week, 60 min per session, via telephone)								Interpersonal-level (Duration: once a week, 60 min per session, via telephone)								Community-level (Duration: once every 2 weeks, 2 h per session, in-person)							
Fourth-arm	**Education-only control group**																							
				X				X				X				X				X				X

X—monthly messages on healthy diet tips, recognizing warning signs of health, understanding dementia, fall prevention tips, health messages, and anti-fraud tips via text, graphics or voice recordings will be sent out.

Participants will receive the individual-level intervention for 2 months, comprising weekly 60-min telephone sessions. This is followed by 4 months of weekly 15-min maintenance sessions delivered via the telephone to reinforce key skills and ensure consistency in intervention duration across arms.

- Second-arm (individual- + interpersonal-level):

Participants will receive the individual-level intervention in the first 2 months followed by the interpersonal-level intervention in the next 2 months, each delivered as weekly 60-min telephone sessions. In the remaining 2 months, participants will receive weekly 15-min maintenance sessions delivered through telephone to consolidate learning from both components.

- Third-arm (individual- + interpersonal- + community-level):

Participants will receive the individual-level (2 months) and interpersonal-level (2 months) interventions as described above, followed by a community-level intervention (2 months) consisting of four in-person, community-led sessions (approximately 2 h each). No maintenance sessions will be provided in this arm to avoid overburden for participants.

- Forth-arm (education-only control):

Participants will receive SMS monthly messages via text, graphics (e.g., posters), or voice recordings for a period of six months.

All intervention sessions are delivered by trained volunteers, supervised by the research team. Volunteers are used because they enable scalable, cost-efficient delivery, and reflect real-world implementation conditions. Detailed session content for each component can be found in Supplementary Tables 1–3.

#### Content

##### Individual-level

The individual-level intervention combines a mindfulness-based approach and cognitive behavioral therapy (CBT) principles. The mindfulness component is adapted from the smartphone-delivered intervention (27) and previously validated among older adults in Hong Kong (29, 30, 56, 96). The CBT component is adapted from SOLUS-D, an evidence-based interactive self-help programme designed to reduce loneliness (32, 57). Participants will receive eight weekly 60-min sessions delivered over the telephone by trained volunteers. Session content includes body awareness and present-moment awareness, emotional awareness and mind–body labelling, relaxation techniques and sensory discrimination, deepening awareness and accepting feelings, exploring intrinsic needs, understanding the interconnection between thought patterns and emotions, overcoming negative thinking, and cultivating a positive mindset. Brief maintenance sessions will reinforce intervention content through guided exercises and supporting videos.

##### Interpersonal-level

The interpersonal-level intervention will adapt content from Group 4 Health (G4H), a psychosocial programme designed to strengthen social group identification and belonging (39). G4H will also be adapted for telephone delivery, while preserving its core theoretical mechanisms through structured reflection, guided exercises, and goal-settings. Participants will engage in eight weekly 60-min sessions delivered via telephone by trained volunteers. Sessions focus on understanding the benefits of social connections, exploring self and community, examining interpersonal networks in daily life, managing and improving interpersonal relationships, expanding social groups, and ongoing practice and review of social goals and resources. Maintenance sessions will reinforce the intervention content through guided practice and supporting materials.

##### Community-level

The community-level intervention will adapt concepts from Neighbours Every Day, a community-based initiative that promotes neighbourhood cohesion through inclusive and socially engaging activities (43). Participants will attend four weekly 2-h in-person sessions over 2 months, held at accessible community venues. Each session will accommodate up to 42 participants, with structured subgroup activities (up to six participants) to encourage deeper engagement. The intervention aims to help participants build neighbourhood connections, get to know each other, strengthen mutual support, enhance community bonds, foster a sense of belonging, create a cohesive community, and establish trustworthy relationships. No maintenance is included to minimize participant burden as the intervention is designed to promote community engagement beyond formal sessions.

##### Education-only control

Participants in the education control group will receive monthly health-related SMS messages over a six-month period, serving as an attention control without introducing active psychosocial or therapeutic elements related to loneliness. Messages include topics such as healthy diet, fall prevention, dementia awareness, general health information, and anti-fraud tips. Therapeutic effects are minimized by avoiding content related to emotional regulation, social connection, or behavioral change techniques. For participants without smartphones, identical content will be delivered via email or postal mail.

#### Feasibility and retention strategies

To reduce fatigue and attrition, the intervention is delivered sequentially, with each level standardized to a two-month period. Telephone delivery of the individual- and interpersonal-level components aim to minimize mobility and scheduling barriers, with sessions arranged according to participants’ availability, with reminder calls provided by the research team. Based on prior experience (58), missed sessions are expected to be minimal. In the event if happens, sessions will be rescheduled within the intervention period and attendance will be documented. The community-level component is limited to four socially engaging, event-based sessions to reduce burden while promoting connection.

### Interventionists

All interventions will be delivered by trained volunteers, primarily retired individuals aged 50 to 70 years, who have completed at least 3 years of secondary education, report no significant physical or mental health issues or cognitive deficits, and are not employed full-time. Each eligible volunteer will be required to complete six weekly 2-h training sessions, led by trained social workers or mindfulness practitioners, depending on the intervention assigned. These sessions will include lectures, reading materials, and structured role plays to provide comprehensive preparation. Prior to intervention delivery, competency assessment will be conducted using observed role-play and standardized evaluation criteria (i.e., content delivery, protocol adherence). Each volunteer will deliver the intervention to one or two participants at a time, allowing for individualized attention and manageable workload. All volunteers will also receive a reimbursement in the form of supermarket vouchers.

#### Quality assurance

To ensure consistency in intervention delivery, standardized training materials, manuals, and recorded training sessions will be utilized. Ongoing supervision will be conducted weekly via group supervision meetings, monthly interventionists conferences, and continuous access to the research team for consultation. At least 20% of intervention sessions will be randomly selected for fidelity review using a structured intervention fidelity checklist documenting session duration, content coverage, participant engagement, and difficulties encountered.

Volunteers will also submit recordings of their initial sessions for review and feedback. Volunteers demonstrating difficulties or protocol deviations will receive targeted feedback, refresher training, or one-to-one supervision. Mid-project booster sessions will be conducted to reinforce competencies and adherence to intervention protocols. For the community-level intervention, an independent researcher will observe activities, document field notes, and report to the research team to ensure protocol adherence.

### Measures

All outcome measures are locally validated and have been selected to capture the primary outcome, secondary outcomes and potential mechanisms of change. All outcome assessments will be conducted face-to-face at the participant’s preferred time and location across baseline (T0), as well as at 2 months (T1), 4 months (T2), and 6 months (T3) from baseline, and at 12 months (T4) and 24 months (T5) after the completion of the intervention. To minimize participant burden, outcome measures are prioritized and staggered across timepoints (see Table 2). All assessments will be administered by experienced, independent interviewers blinded to intervention allocation. All interviewers will undergo rigorous training involving comprehensive familiarization with the measurement tools, clinical vignettes, and on-site practice to achieve and maintain high inter-rater and intra-rater reliability (ICC >0.9) throughout the study.

#### Primary outcome

Loneliness will be assessed by the 20-item Revised UCLA Loneliness Scale (UCLA-LS) (59, 60), where participants will rate each item based on the frequency they experience feelings of loneliness, using a 4-point Likert scale ranging from 1 = Never to 4 = Always. Total scores range from 20 to 80, with higher scores indicating greater levels of loneliness.

#### Secondary outcomes

Loneliness will also be measured by the 6-item De Jong Gierveld Loneliness Scale (DJGL) (61, 62). Participants will rate their agreement with each statement on a 3-point Likert scale ranging from 1 = Yes to 0 = No. DJGL includes two subscales: emotional loneliness (3 items) and social loneliness (3 items). Total scores range from 0 to 6, with subscale score ranging from 0 to 3. A high score indicates higher levels of loneliness.

Sleep quality will be assessed by the 19-item Pittsburgh Sleep Quality Index (PSQI) (63, 64). PSQI evaluates seven components of sleep: sleep quality, sleep latency, sleep duration, sleep efficiency, sleep disturbance, use of sleep medication, and daytime dysfunction. Participants will rate each item on a 4-point Likert scale ranging from 0 = No difficulty to 3 = Severe difficulty. Total scores range from 0 to 21, with higher score indicating worse sleep quality.

Life satisfaction will be assessed by the Satisfaction with Life Scale (SWLS) (65, 66). Participants will rate their agreement with each statement related to subjective well-being and life satisfaction on a 7-point Likert scale ranging from 1 = Strongly disagree to 7 = Strongly agree. Total scores range from 5 to 35, with a higher score indicating greater life satisfaction.

Perceived stress will be assessed by the 14-item Perceived Stress Scale (PSS) (67, 68). Participants will rate the frequency with which they experienced each feeling in the past month on a 5-point Likert scale ranging from 0 = Never to 4 = Very Often. Some items will need to be reverse coded before summing. Total scores range from 0 to 56, with a higher score indicating more stress.

Mental health will be assessed by the 14-item Hospital Anxiety and Depression Scale (HADS) (69, 70). Participants will rate the intensity of each symptom on a 4-point Likert scale ranging from 0 = Not at all to 3 = Most of the time. HADS consists of two subscales: anxiety (7 items) and depression (7 items). Total scores range from 0 to 42, with subscale score ranging from 0 to 21. A higher score indicates greater severity of anxiety or depression.

Cognitive functioning will be assessed by the Montreal Cognitive Assessment (MoCA) (71, 72). MoCA consists of seven cognitive domains: executive functioning, naming, memory, attention, language, abstraction, and orientation. Participants will complete 11 tasks, and total scores range from 0 to 30, with a higher score indicating better cognitive performance.

Physical heath will be assessed in five ways: Barthel Index of Independence in Activities of Daily Living (ADL) (73), Lawton Instrumental Activities of Daily Living Scale (IADL) (74, 75), 18-item Physical Symptoms Inventory (PSI) (76, 77), Charlson Comorbidity Index (CCI) (78), and 6-Minute Walking Test (6MWT-CHN) (79, 80). For ADL, participants will rate their current level of ability across 10 items assessing activities of daily living on a scale of 0 = dependent to 5/10 = independent. Total scores range from 0 to 100, with higher scores indicating greater independence. For IADL, participants will rate their ability in performing 8 specific tasks on a scale of 0 = dependent to 1 = independent. Total scores range from 0 to 8, with higher scores indicating greater independence. For PSI, participants will rate the presence of each symptom in the past 30 days on a 5-point Likert scale ranging from 0 = Never to 4 = Often. Total scores range from 0 to 72, with a higher score indicating a greater number and frequency of reported symptoms. For CCI, participants will indicate the presence of a list of 16 comorbid conditions (e.g., heart failure, diabetes, chronic pulmonary disease, lymphoma). Each condition is assigned a weighted score based on its associated risk of mortality, leading to a total score ranging from 0 to 37. Higher scores indicate a greater burden of comorbidities and predict a higher risk of mortality. For 6MWT-CHN, participants will be asked to walk the furthest distance possible in 6 min; they may slow down, stop, and rest during the test, but should resume walking as soon as they are able. Before and immediately after the walking test, participants will rate their level of breathlessness using the Borg scale from 0 = No breathlessness to 10 = Maximal breathlessness. The total distance walked in 6 min will be recorded as well.

#### Mediators

Mindfulness will be assessed by the 15-item Mindfulness Attention Awareness Scale (MAAS) (81). Participants will rate the frequency of each experience on a 6-point Likert scale ranging from 1 = Almost always to 6 = Almost never. A mean score across all items will be calculated, with higher scores indicating higher levels of dispositional mindfulness.

Self-compassion will be assessed by the 12-item Self-Compassion Scale-Short Form (SCS-SF) (82, 83). SCS-SF comprises six subscales: self-kindness, self-judgment, humanity, isolation, mindfulness, and over-identification. Participants will rate how often they behave in the manner described by each statement on a 5-point Likert scale ranging from 1 = Almost never to 5 = Almost always. A mean will be calculated for each subscale and overall scale, with higher scores indicating higher levels of self-compassion.

Thought patterns will be assessed by the Emotion Regulation Questionnaire-Cognitive Reappraisal subscale (ERQ-CR) (84, 85). Participants will rate the extent to which they engage in cognitive reappraisal through their speech, gesture, or behavior on a 7-point Likert scale ranging from 1 = Strongly disagree to 7 = Strongly agree. A mean score will be calculated, with higher scores indicating greater use of cognitive reappraisal as an emotion regulation strategy.

Social support will be assessed by the 12-item Multidimensional Scale of Perceived Social Support (MSPSS) (86, 87). Participants will rate their perceived support from three: family, friends, and significant others on a 7-point Likert scale ranging from 1 = Strongly disagree to 7 = Strongly agree. Total scores range from 12 to 84, with subscale score ranging from 4 to 28. A higher score indicates higher levels of perceived support.

Social network will be assessed by the 6-item Lubben Social Network Scale (LSNS-6) (88, 89). Participants will rate how many family members and friends they can engage with in terms of frequency of contact, closeness for discussing private matters, and availability for help on a 6-point Likert scale ranging from 0 = None to 5 = 9 or more. Total scores range from 0 to 30, with a higher score indicating greater social engagement and lower risk of social isolation.

Neighbourhood collective efficacy and identity will be assessed by the modified 8-item Neighbourhood Collective Efficacy Scale (NCE) (90, 91). Participants will rate statements focusing on social cohesion and informal social control in their neighbourhood on a 5-point Likert scale ranging from 1 = Strongly agree to 5 = Strongly disagree. A mean score will be calculated, with higher scores indicating greater social control, cohesion, and trust.

#### Cost-effectiveness

Quality of life will be assessed by the EuroQol 5-Dimension 5-Level Instrument (EQ-5D-5L) (92, 93). EQ-5D-5L consists of two parts: descriptive system (EQ-5D) and a Visual Analogue Scale (EQ-VAS). In the descriptive system, participants will rate their health status across five dimensions: mobility, self-care, usual activities, pain/discomfort, and anxiety/depression on a 5-point Likert scale ranging from 0 = No problem to 5 = Unable to perform. For the EQ-VAS, participants will rate their overall health status on the day of assessment from 0 = Worst health to 100 = Best health.

Healthcare service usage will be evaluated using a self-designed scale. Participants will report the frequency of their use of outpatient medical treatment, accident and emergency treatment, and inpatient medical treatment over the past 3 months at both public and private facilities. Frequencies will be rated on a scale from 0 = none to 3 = three times or more.

#### Demographics

The following demographic characteristics will be collected: gender, age, marital status, number of children, living arrangements with family members, highest level of educational attainment, economic activity status, utilization of senior citizen centres or senior citizen services, use of community or home care services, and household income in the past month.

### Process evaluation

The process evaluation will be guided by the Reach, Effectiveness, Adoption, Implementation, Maintenance (RE-AIM) framework (94). To assess the quality and participant satisfaction, participants will complete a treatment evaluation inventory and rate their satisfaction with their interventionists.

#### Focus group discussion

A subset of participants will be invited to join the focus group discussions conducted separately for each intervention group within 1 week after completing the intervention. During these discussions, participants will respond to a semi-structured, open-ended questions centered on 4 key areas: (i) their reasons and expectations for participating in the study, (ii) their feelings, favourite or least favourite parts, perceived helpfulness, and challenges related to the intervention content, (iii) any positive or negative impacts experienced from the intervention towards their mental health, thought patterns, and social connections, and (iv) their satisfaction with the intervention content, the interventionist, or suggestions for improvements.

### Planned statistical analysis

Descriptive statistics will be used to summarize baseline characteristics of the participants within each arm. An intention-to-treat approach will be adopted. Missing values will not be replaced because mixed-effects models can accommodate participants with at least one outcome measurement under the assumption that data are missing at random. All analyses will be appropriately accounted for the cluster randomized controlled trial (cRCT) design, recognizing that participants are nested within clusters and outcomes within clusters may be correlated. To assess intervention effects of different arms on primary and secondary outcomes, generalized linear mixed models (GLMM) with random intercepts for clusters, fixed effects for time points (i.e., T0, T1, T2, T3, T4, T5), group (using the control group as the reference), and their interaction terms will be utilized. In addition to GLMM, generalized estimating equations (GEE) with robust standard errors and an exchangeable working correlation structure will be used for sensitivity analysis, with small sample corrections applied as needed to control the rate of type I error. To ensure robustness, results from GLMM and GEE approaches will be compared, with multiple testing corrections such as Bonferroni adjustments applied as appropriate. Sensitivity analyses will include per-protocol and complete case approaches to evaluate the influence of adherence and missing data. Mediation analyses of psychosocial and other potential mediators will employ multilevel modelling techniques suited for clustered data. Statistical significance was defined as *p* < 0.05. All statistical modelling will be conducted using SPSS version 29.0, which provides robust tools for multilevel mixed-effects modelling including GLMM and GEE, with options for controlling cluster effects and covariate adjustment.

For the focus group discussions, all recordings will be transcribed verbatim by a research assistant. Two independent researchers will then conduct a thematic analysis of the transcripts using NVivo version 15 (95). The research team will review and agree on the identified codes, organizing them into main categories and subcategories. These categories may be condensed and reorganized as needed. The team will ensure consistency, reach consensus on the meaning and structure of the categories, and develop a finalized list. Ultimately, a set of categorized themes with supporting verbatim quotes will be produced to highlight the major strengths and limitations of the interventions and to inform improvements for their future implementation.

To evaluate the cost-effectiveness of the multi-level intervention compared to the control group, a trial-based economic evaluation will be conducted. Health outcomes will be measured as quality-adjusted life years (QALYs), calculated using the standard area-under-the-curve method based on individual EQ-5D-5L responses over the study period. Resource utilization will be estimated from the provider’s perspective, and costs for the intervention, outpatient care, emergency department visits, and inpatient care will be calculated using a bottom-up costing approach. The multi-level intervention will be considered dominant if it achieves higher QALYs at a lower cost than the control group. If it results in higher QALYs but also higher costs, the incremental cost-effectiveness ratio (ICER), defined as the difference in cost divided by the difference in QALYs (ΔCost/ΔQALYs), will be calculated. The intervention will be deemed cost-effective if it is dominant or if the ICER falls below the accepted willingness-to-pay threshold. The probability of the intervention to be accepted as cost-effective will be evaluated by probabilistic sensitivity analysis over the uncertainties of all parameters of costs and QALYs as well as variation of willingness-to-pay threshold from zero to 3× gross domestic product per capita in Hong Kong, presented as acceptability curves.

### Ethical considerations and dissemination

The trial has been approved by the Human Research Ethics Board of The Education University of Hong Kong (2023-2024-0624) and is prospectively registered with ClinicalTrials.gov (NCT07123064). Prior to enrolment, all participants will receive detailed written information about the study and provide written informed consent. Signed informed consent will be obtained before any study procedures commence. Recruitment will adhere to the principle of voluntariness, and all participants’ data will be kept confidential. Participants will receive supermarket vouchers upon completion of the intervention and assessments. If a participant decides to withdraw from the study, their wishes will be complied with, and reasons for the withdrawal will be sought. Findings from this trial will be widely disseminated through press conferences, scientific conferences, peer-reviewed journals, and stakeholder organizations.

## Results

The preparation phase, including the finalization of the intervention design, assessment tools, and training of research personnel, took place from June to November 2025. Recruitment and enrolment of participants, along with volunteer training, are planned to begin in March 2026. Baseline assessments are scheduled for April 2026, with intervention delivery to follow. Follow-up assessments will continue until March 2029.

## Discussion

Loneliness has increasingly been recognized as a major public health concern before, during, and after the COVID-19 pandemic. Although most existing interventions have focused primarily on the individual-level strategies, there remains a pressing need for more comprehensive, multilevel approaches capable of addressing the complex and interconnected determinants of loneliness. Building on our prior individual-level work (29, 30, 56, 58), the present study introduces a novel three-tier intervention for community-dwelling older adults experiencing loneliness and poverty (96). The primary aim is to develop, implement, and evaluate the effectiveness of three intervention intensities (i.e., individual-level only; individual- + interpersonal-level; and individual- + interpersonal- + community-level) on loneliness, assessed with the UCLA-LS and DJGL scales at 2-, 4-, 6-, 12-, and 24-month follow-ups, and to determine the most effective and sustainable configuration. A broad set of secondary outcomes—including physical and mental health indicators, cognitive functioning, and cost-effectiveness—will also be assessed over 24 months to capture the multidimensional impact and economic feasibility of these approaches.

A major strength of this study is its multidisciplinary team, bringing together expertise in intervention development, contextual understanding, and the design and execution of large RCTs. The involvement of key community partners (Oxfam Hong Kong, the Society for Community Organization, and the Research Centre for Gerontology and Family Studies) is essential for successful implementation. These organizations enhance access to individuals living in poverty, help engage other social service providers, and support the rapid resolution of challenges encountered during implementation. Another important strength is the use of telephone-based delivery and trained volunteers. This strategy leverages existing community resources, enhances scalability, and provides a cost-effective method for reaching older adults who may face mobility, financial, or digital barriers. Evidence from previous studies (29, 30) demonstrates that such approaches can enhance both operational sustainability and participant engagement (96).

Despite these strengths, the study presents several potential challenges. First, participants in the more intensive arms (i.e., individual- + interpersonal-level; and individual- + interpersonal- + community-level) may experience adherence difficulties or intervention fatigue. To mitigate these issues, flexible scheduling will be offered to accommodate participants’ energy levels, health conditions, and personal circumstances. Intervention intensity will also be adjusted to individual needs without compromising the core therapeutic components. For example, volunteers may devote additional time to selected topics, provide tailored feedback or supplementary materials in coordination with the project team, and employ a variety of engagement techniques to support diverse learning styles. A second limitation concerns the risk of participant attrition, particularly given the extended 24-month follow-up period. To address this, monthly check-ins and milestone-based financial incentives will be provided after the intervention period to encourage continued participation, maintain rapport, and reduce dropout rates.

Overall, this study is expected to generate rigorous evidence regarding the effectiveness of multilevel interventions to reduce loneliness among older adults, especially those living with poverty and intersecting social vulnerabilities. By integrating individual, interpersonal, and community components—and leveraging social capital through trained volunteers and technology-supported communication—the intervention is designed to be effective, accessible, affordable, and scalable. The longitudinal design enables the examination of both short- and long-term outcomes, offering insights into sustainable best practices. The findings are anticipated to guide policymakers, practitioners, and community organizations in developing programmes that strengthen social connectedness, improve quality of life, and promote healthy aging at a population level.
