# Supplementary material for: Study Protocol: Connecting older adults through multi-level programmes for alleviating loneliness in Hong Kong older adults (COMPASS-HOA)
Source: Front Public Health. 2026 Feb 23;14:1741422. doi: 10.3389/fpubh.2026.1741422 (PMC12968008; doi:10.3389/fpubh.2026.1741422)
Supplement: Supplementary file 1 [file Data_Sheet_1.docx]

**Supplementary Material**

**Supplementary Table 1: Session content of individual-level intervention**

| **Session** | **Topic** |
| --- | --- |
| 1 | Nature of loneliness and introduction to mindfulness: Body awareness and present-moment experience |
| 2 | Emotional awareness and mind-body labelling: Understanding and tagging emotions |
| 3 | Relaxation techniques and sensory discrimination: Enhancing inner peace |
| 4 | Deepening awareness and accepting feelings: Embracing emotional experience |
| 5 | Revisiting loneliness and personal values: Exploring intrinsic needs |
| 6 | Loneliness and cognition: The interconnection between though patterns and emotions |
| 7 | Identifying and analysing of thought traps: Overcoming negative thinking |
| 8 | Challenging thoughts and future planning: Cultivating positive mindset |

**Supplementary Table 2: Session content of interpersonal-level intervention**

| **Session** | **Topic** |
| --- | --- |
| 1 | Understanding the benefits of social connections |
| 2 | Understanding social identity map tool |
| 3 | Creating personal social identity map |
| 4 | Managing and improving interpersonal relationships |
| 5 | Understanding social group changes and challenges |
| 6 | Managing challenging group |
| 7 | Expanding new social groups |
| 8 | Summary and moving forward: Continuous practice and review |

**Supplementary Table 3: Session content of community-level intervention**

| **Session** | **Topic** |
| --- | --- |
| 1 | Building neighbourhood connections and getting to know each other |
| 2 | Healthy aging and neighbourhood care: Strengthening neighbourhood mutual support and enhancing community bonds |
| 3 | Vibrant exploration and thriving community: Building our community together and fostering belonging |
| 4 | Connecting the dots: Building trustworthy relationships  Programme wrap-up |
